# Diagnostic usefulness of the spot urine sodium/potassium ratio in cirrhotic patients with ascites

**DOI:** 10.1371/journal.pone.0253886

**Published:** 2021-06-24

**Authors:** Jin Wook Lee, Jae Seok Hwang, Woo Jin Chung, Heon Ju Lee, Jung Gil Park, Chang Hyeong Lee, Byung Seok Kim, Jeong Eun Song, Young Oh Kweon, Won Young Tak, Soo Young Park, Se Young Jang, Jeong Ill Suh, Byoung Kuk Jang

**Affiliations:** 1 Department of Internal Medicine, Keimyung University School of Medicine, Daegu, South Korea; 2 Department of Internal Medicine, Bokwang Hospital, Daegu, South Korea; 3 Department of Internal Medicine, Yeungnam University College of Medicine, Daegu, South Korea; 4 Department of Internal Medicine, Daegu Catholic University School of Medicine, Daegu, South Korea; 5 Department of Internal Medicine, School of Medicine, Kyungpook National University Hospital, Kyungpook National University, Daegu, South Korea; 6 Department of Internal Medicine, Dongguk University College of Medicine, Gyeongju, South Korea; University of Florida, UNITED STATES

## Abstract

**Background and aims:**

The low-salt diet is considered important for control of ascites in cirrhotic patients. To validate whether the spot urine sodium (Na)/potassium (K) ratio could replace 24-h urine Na (uNa) excretion in assessing low-salt diet compliance.

**Methods:**

We prospectively studied 175 patients. 24-h urine collection and spot urine collection were performed. Subsequently, 24-h uNa, urine creatinine (uCr), and spot urine Na and K were assessed. A complete urine collection was confirmed based on 24-h uCr excretion levels of 15mg/kg/day for men and 10mg/kg/day for women. The area under the receiver operating characteristic (AUROC) curve analysis was performed to evaluate the feasibility of spot urine Na/K ratio in predicting 24-h uNa greater than 78mmol/day.

**Results:**

Out of 175 patients, 24-h urine samples were completely collected in 57 patients only. Moreover, urine samples were not completely collected in 118 patients because their 24-h uCr excretion level was less than the established criteria. In complete urine collection group, AUROC curve for spot urine Na/K ratio in predicting 24-h uNa greater than 78mmol/day was 0.874±0.051 (P<0.001). In the incomplete urine collection group, the AUROC was 0.832±0.039 (P<0.001). In complete urine collection group, the classical cutoff value greater than 1.0 of spot urine Na/K ratio showed 90.9% sensitivity and 56.0% specificity.

**Conclusions:**

The spot urine Na/K ratio reflects 24-h uNa, but the AUROC value obtained in this study is lower than that of a previous study. Considered the large number of patients with incomplete urine collection, validating 24-h complete urine collection criteria is necessary.

## Introduction

Ascites is a common complication in liver cirrhotic patients and is observed in approximately 50% of patients with compensated cirrhosis in 10 years [1]. Moreover, ascites is the most common complication observed in hospitalized cirrhotic patients [2]. It is known that 15% and 44% of patients with ascites die within 1 and 5 years, respectively [3].

Ascites is observed in cirrhosis due to portal hypertension and hepatic insufficiency. Subsequently, retention of sodium (Na) and water in the kidney is observed, with the renin-angiotensin-aldosterone system playing an important role [4, 5]. In the treatment of ascites with liver cirrhosis caused by this mechanism, it is important to initially treat the underlying liver disease, to limit salt intake, and to take diuretics. In general, low-salt diets help control ascites and shorten hospital stays [6], and the recommended daily salt intake in cirrhotic patients is less than 5 g (Na, 2 g [88 mmol]).

Dietary salt restriction is the main treatment for cirrhotic patients with ascites. However, it has been reported that only 10%–15% of patients control their ascites by dietary salt restrictions. Therefore, additional diuretics are required to control the ascites in cirrhotic patients [7]. In the underlying assumptions, non-urinary Na excretion is known to be less than 10 mmol/day [8]; hence, 24-h urine Na excretion in patients taking 5 g of salt does not exceed 78 mmol. In patients taking diuretics combined with a low-salt diet, with urine Na excretion greater than 78 mmol, fluid weight loss is expected. In patients with insufficient weight loss even with diuretic use, with greater than 78 mmol of 24-h urine Na excretion, a low-salt diet is considered insufficient. Thus, strict low-salt dietary training is required, and if the 24-h urine Na excretion is less than 78 mmol, increasing the diuretic dose is required because diuretics are insufficiently effective [9]. In some cases, ascites is not controlled even with high-dose diuretic treatment and salt intake restrictions. Subsequently, it is defined as diuretic-resistant ascites, and patients with this type of ascites should consider secondary treatments such as repeated paracentesis, transjugular intrahepatic portosystemic shunt, or liver transplantation [7, 10, 11]. In monitoring ascites, 24-h urine Na excretion can be measured to assess whether a low-salt diet is maintained and to evaluate the response of the diuretic dose [9, 12]. Although the 24-h urine Na test is important to control ascites, 24-h urine collection is time-consuming for patients, and frequently, in this test, urine samples are not collected correctly. With the inadequate urine collection, interpreting the data in clinical practice is considered difficult. Therefore, it is known that the spot urine Na/potassium (K) ratio can be alternatively measured [13]. However, whether the 24-h urine Na excretion can be replaced with the spot urine Na/K ratio has not been fully validated yet. Hence, this study aimed to validate whether the spot urine Na/K ratio could replace 24-h urine Na excretion in assessing low-salt diet compliance.

## Methods

### Subjects

This study was approved by the Institutional Review Board of Keimyung University Dongsan Hospital, Daegu, Korea (DSMC 2016-04-043). Patients provided informed written consent after the study protocols and objectives were comprehensively explained to the patients (ClinicalTrials.gov Identifier: NCT03263598). The patients who could not provide informed consent for any reason were excluded (e.g., hepatic encephalopathy, dementia). From May 2016 to February 2018, the study was prospectively conducted with 192 recruited patients with liver cirrhosis and ascites who visited the following five tertiary university hospitals: Keimyung University Dongsan Hospital, Kyungpook National University Hospital, Daegu Catholic University Hospital, Dongguk University Hospital, and Yeungnam University Hospital. Patients who needed blood and urine tests for ascites evaluation and ascites control were recruited. Liver cirrhosis was diagnosed based on compatible clinical, biochemical, and radiologic findings (abdominal computed tomography [CT] and abdominal ultrasonography). Patients with systemic infections or sepsis, with underlying kidney disease, and with serum creatinine (Cr) level greater than 1.5 mg/dL and patients treated with malignancy, except hepatocellular carcinoma, were excluded.

### Study design

The study protocol was as follows. All patients underwent routine blood test, and their viral hepatitis markers were also assessed. During this study, patients’ dietary salt intake was limited to less than 5 g per day. Water was not strictly restricted. Moreover, excessive consumption of fruits and vegetables was prohibited. Diuretics were administered in conventional dose regimens once a day or twice a day. Patients were given a urine collection cup, a urine container, and written instructions for urine collection. Each subject was instructed to collect a 24-h urine sample the day before presenting to the hospital. Random urine samples were obtained two to nine hours after taking diuretics in the morning for the measurement of the spot urine Na/K ratio (AM10-PM5). Urine samples were sent to the laboratory to assess 24-h urine Na, 24-h urine Cr, and spot urine Na and K. The association between 24-h urine Na excretion and the spot urine Na/K ratio was evaluated. A complete 24-h urine collection was confirmed based on 24-h Cr excretion levels of 15 mg/kg/day for men and 10 mg/kg/day for women [14]. The presence of sarcopenia in patients was confirmed by measuring psoas muscle thickness per height (PMTH). PMTH was determined by measuring transverse psoas muscle thickness at the umbilical level of patients who underwent abdominal CT imaging within 3 months before and after the study participation [15].

### Statistical analyses

In this study, 188 patients were needed as the sample size required to estimate proportions with sensitivity of 90%, specificity of 80%, dropout rate of 40% and α of 0.05 was determined [16]. All data were analyzed using the International Business Machines Corporation (IBM®) Statistical Package for the Social Sciences® version 21 software (IBM Corp., Armonk, NY, USA). Numerical variables are expressed as mean and standard deviation. Continuous variables were compared using Student’s t-test or Mann-Whitney U test. The Chi-squared test or Fisher’s exact test was used to compare the nominal variables between the two groups. Diagnostic accuracy of the spot urine Na/K ratio was analyzed by estimating the area under the receiver operating characteristic (AUROC) curve and by calculating the sensitivity, specificity, positive predictive value (PPV), and negative predictive value (NPV).

## Results

**Fig 1:**
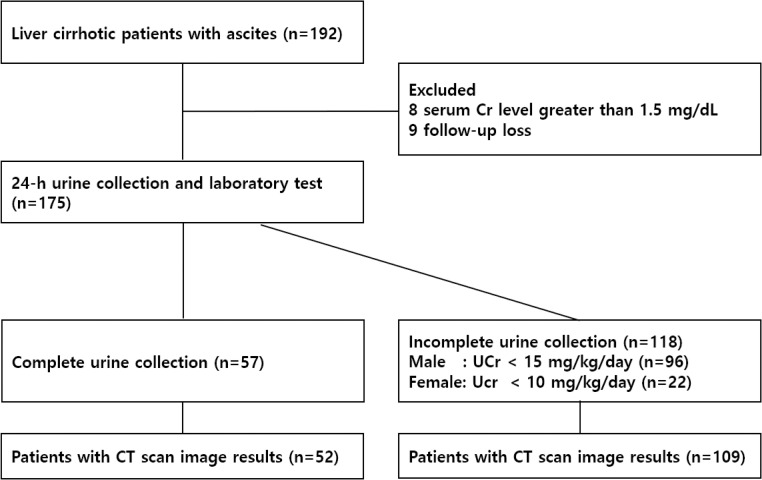
Flow diagram of patient’s participation in the study.

A total of 192 patients were enrolled, and eight patients with serum Cr level greater than 1.5 mg/dL were excluded. Nine patients who were not followed up were excluded, and 175 patients met the inclusion criteria. Only 57 of the 175 patients met the 24-h urine Cr excretion level criteria as a basis for complete urine collection. The 24-h urine collections from the other 118 patients did not meet the predetermined criteria for a complete collection (Fig 1).

### Clinical characteristics and laboratory test results

The clinical characteristics and laboratory test results of patients in the complete urine collection group were as follows. The mean age and standard deviation was 53.6 ± 12.8 years, the proportion of male among the patients was 71.9%, and the most common etiology for cirrhosis was alcohol consumption (64.9%). The proportions of patients with hepatitises B and C were 21.1% and 8.8%, respectively. Furthermore, according to the Child-Pugh classification, the proportions of patients diagnosed with Child-Pugh A, B, and C were 17.5%, 52.6%, and 29.8%, respectively. The mean Model for End-Stage Liver Disease (MELD) score was 11.5 ± 5.8. Moreover, 80.7% of patients took furosemide and spironolactone in combination, 14.0% of patients did not take diuretics. All demographic, clinical, and laboratory data are summarized in Table 1. In the incomplete urine collection group, the mean age and standard deviation was 56.5 ± 10.6 years, the proportion of male among the patients was 81.4%, and the most common etiology of cirrhosis was alcohol consumption (62.7%). The proportions of patients with hepatitises B and C were 22.9% and 5.9%, respectively. Moreover, according to the Child-Pugh classification, the proportions of patients diagnosed with Child-Pugh A, B, and C were 11.0%, 57.6%, and 31.4%, respectively. The mean MELD score was 11.8 ± 4.9. Furthermore, 83.9% of patients took furosemide and spironolactone in combination, 12.7% of patients did not take diuretics. Between the two groups, there was no statistically significant difference in baseline characteristics, etiology of liver cirrhosis, distribution of Child-Pugh classification, MELD score, and the type of diuretics use. Additionally, the laboratory values of hemoglobin, platelet, serum albumin, serum bilirubin, aspartate transaminase, alanine transaminase, blood urea nitrogen, Cr, serum Na, serum K, and prothrombin time international normalized ratio were insignificantly different between the two groups (Table 1).

**Table 1:** Patients’ clinical characteristics and laboratory test results.

Characteristics	All patients	Complete urine collection	Incomplete urine collection	*P*-value
	N = 175	N = 57	N = 118	
Age (years)	55.5 ± 11.4	53.6 ± 12.8	56.5 ± 10.6	0.158
Sex				0.156
Male	137 (78.3%)	41 (71.9%)	96 (81.4%)	
Female	38 (21.7%)	16 (28.1%)	22 (18.6%)	
Etiology of LC				0.649
Alcohol	111 (63.4%)	37 (64.9%)	74 (62.7%)	
Hepatitis B	39 (22.3%)	12 (21.0%)	27 (22.9%)	
Hepatitis C	12 (6.9%)	5 (8.8%)	7 (5.9%)	
AIH	4 (2.3%)	0 (0%)	4 (3.4%)	
Cryptogenic	9 (5.1%)	3 (5.3%)	6 (5.1%)	
Child-Pugh class				0.485
	23 (13.1%)	10 (17.5%)	13 (11.0%)	
B	98 (56.0%)	30 (52.7%)	68 (57.6%)	
C	54 (30.9%)	17 (29.8%)	37 (31.4%)	
MELD score	11.7 ± 5.2	11.5 ± 5.8	11.8 ± 4.9	0.715
Laboratory finding				
Diuretics				0.804
Furosemide and spironolactone	145 (82.9%)	46 (80.7%)	99 (83.9%)	
Furosemide alone	7 (4.0%)	3 (5.3%)	4 (3.4%)	
No diuretics	23 (13.1%)	8 (14.0%)	15 (12.7%)	
Hemoglobin (g/dL)	10.7 ± 1.7	10.4 ± 1.7	10.9 ± 1.7	0.253
Platelet (/uL)	115,700 ± 65,700	105,130 ± 49,070	121,000 ± 72,200	0.092
Serum albumin (g/dL)	2.9 ± 0.7	2.9 ± 0.6	2.9 ± 0.7	0.807
Serum bilirubin (mg/dL)	3.6 ± 4.4	3.2 ± 3.7	3.8 ± 4.7	0.410
AST (U/L)	89 ± 130	99.3 ± 203	84.3 ± 77.3	0.439
ALT (U/L)	48 ± 170	73.3 ± 297	35.8 ± 34.8	0.326
BUN (mg/dL)	14.7 ± 8.2	15.2 ± 8.2	14.5 ± 8.3	0.519
Cr (mg/dL)	0.8 ± 0.2	0.8 ± 0.2	0.8 ± 0.2	0.517
Serum Na (mmol/L)	135.0 ± 7.6	135.4 ± 4.1	134.9 ± 8.9	0.650
Serum potassium (mmol/L)	4.1 ± 0.6	4.0 ± 0.6	4.1 ± 0.6	0.542
PT INR	1.4 ± 0.3	1.5 ± 0.4	1.4 ± 0.2	0.605
24-h urine volume (mL)	1753.2 ± 916.1	2046.8 ± 971.1	1617.7 ± 863.3	0.006
24-h uNa (mmol/d)	106.4 ± 79.7	113.2 ± 84.0	103.0 ± 78.3	0.825
24-h uCr (mg/kg/day)	12.5 ± 4.4	17.0 ± 3.3	10.4 ± 3.1	0.001
24-h uNa>78mmol	102 (58.3%)	32 (56.1%)	70 (59.3%)	0.689
24-h uNa<78mmol	73 (41.7%)	25 (43.9%)	48 (40.7%)	
		Complete urine collection N = 52 (52/57)^a^	Incomplete urine collection N = 109 (109/118)^b^	P
PMTH (mm/m)				
All patients		14.3 ± 4.8	13.0 ± 4.0	0.104
Male		15.4 ± 4.4	13.4 ± 4.0	0.021
Female		11.7 ± 4.7	11.3 ± 3.6	0.807
Sarcopenia by PMTH				
All patients		29/52 (55.8%)	75/109 (68.8%)	0.116
Male		22/36 (61.1%)	68/88 (75.6%)	0.079
Female		7/16 (43.8%)	7/21 (33.3%)	0.733

Values in table are presented as the mean ± standard deviation, or frequency (percentage) for categorical variables. The proportions are presented as percentages for categorical variables.

^a^52 of the 57 patients had computed tomography scan image results.

^b^109 of the 118 patients had computed tomography scan image results.

Abbreviations: LC, liver cirrhosis, AIH, autoimmune hepatitis, MELD, model for endstage liver disease, AST, aspartate aminotransferase, ALT, alanine aminotransferase, BUN, blood urea nitrogen, Cr, creatinine, Na, sodium, PT INR, prothrombin time international normalized ratio, PMTH, psoas muscle thickness per height.

In 24-h urine collection laboratory findings, the complete urine collection group was compared with the incomplete urine collection group. The complete urine collection group had higher mean 24-h urine volume (2046.8 ± 971.1 mL vs. 1617.7 ± 863.3 mL, P = 0.006) and higher mean 24-h urine Cr (17.0 ± 3.3 mg/kg/day vs. 10.4 ± 3.1 mg/kg/day, P = 0.001) than the incomplete urine collection group. There was no significant difference in the mean 24-h urine Na and portion of 24-h urine Na greater than 78 mmol (56.1% vs. 59.3%, P = 0.689) between the two groups (Table 1).

### PMTH and sarcopenia of patients

To confirm sarcopenia, the CT scan results of 52 patients in the complete urine collection group and 109 patients in the incomplete urine collection group were analyzed within 3 months before and after participating in the clinical study. PMTH was significantly higher in men in the complete urine collection group than that in men in the incomplete urine collection group (15.4 ± 4.4 vs. 13.4 ± 4.0, P = 0.021). However, in women, there was no significant difference in PMTH between the two groups (11.7 ± 4.7 vs. 11.3 ± 3.6, P = 0.807). When sarcopenia was diagnosed based on PMTH, the proportion of sarcopenia was higher in men in the incomplete urine collection group than that in men in the complete urine collection group (61.1% vs. 75.6%, P = 0.079) (Table 1).

### AUROC and diagnostic performance of the spot urine Na/K

**Fig 2:**
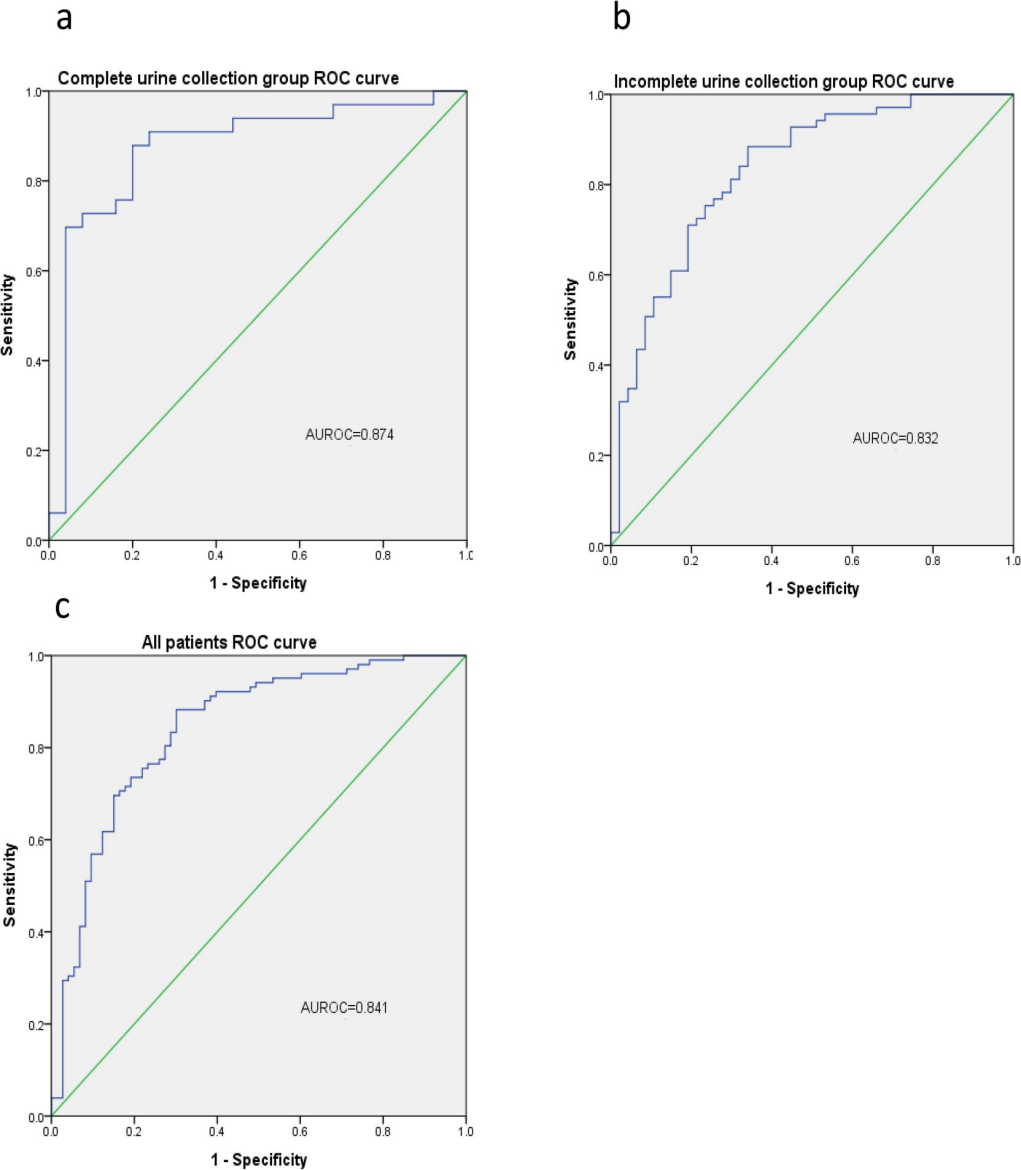
ROC curve of the spot urine sodium (Na)/potassium ratio in predicting 24-h urine Na excretion. a: the complete urine collection group, b: the incomplete urine collection group, c: all patients. Abbreviations: ROC receiver operating characteristic.

The AUROC curve for the spot urine Na/K ratio in predicting 24-h urine Na greater than 78 mmol/day was 0.874 ± 0.051 (P<0.001) in the complete urine collection group. The diagnostic performance of the spot urine Na/K ratio, with a cutoff value of 1.0, in predicting 24-h urine Na greater than 78 mmol/day showed 90.9% sensitivity, 56.0% specificity, 73.2% PPV, and 82.4% NPV. In the incomplete urine collection group, the AUROC curve of the spot urine Na/K ratio in predicting 24-h urine Na greater than 78 mmol/day was 0.832 ± 0.039 (P<0.001) (Fig 2). Furthermore, the diagnostic performance of the spot urine Na/K ratio, with a cutoff value of 1.0, in predicting 24-h urine Na greater than 78 mmol/day showed 95.7% sensitivity, 43.8% specificity, 71.0% PPV, and 87.5% NPV (Table 2). There were no significant differences in the AUROC values, sensitivity, and specificity between the two groups. In all patients, the AUROC value was 0.841 ± 0.031 (P<0.001), with 94.1% sensitivity, 47.9% specificity, 71.6% PPV, and 85.4% NPV when the cutoff value for the spot urine Na/K ratio was 1.0. Table 2 shows in detail the diagnostic performance of the spot urine Na/K ratio in predicting 24-h urine Na greater than 78 mmol/day.

**Table 2:** Sensitivity and specificity of the different spot urine sodium (Na)/potassium ratios in predicting 24-h urine Na excretion.

	Ratio	Sensitivity (%)	Specificity (%)	PPV (%)	NPV (%)
All patients (N = 175)	1	94.1	47.9	71.6	85.4
Na/K ratio	1.5	89.2	63.0	77.1	80.7
	2.0	76.5	75.3	81.3	69.6
	2.5	69.6	84.9	86.6	66.7
Complete urine collection (N = 57)	1	90.9	56.0	73.2	82.4
Na/K ratio	1.5	87.9	80.0	85.3	83.3
	2	75.8	80.0	83.3	71.4
	2.5	69.7	96.0	95.8	70.6
Incomplete urine collection (N = 118)	1	95.7	43.8	71.0	87.5
Na/K ratio	1.5	89.9	54.2	73.8	78.8
	2	76.8	72.9	80.3	68.6
	2.5	69.6	79.2	82.8	64.4

Abbreviations: PPV, positive predictive value, NPV, negative predictive value.

### Analysis by location of urine collection

We analyzed whether the proportion of 24-h complete urine collection differs according to the location of urine collection. When comparing the inpatient urine collection group and the outpatient urine collection group, the mean 24-h urine volumes were 1698.3 ± 923.4 mL in the inpatient urine collection group and 1914.3 ± 884.9 mL in the outpatient urine collection group. There was no significant difference between the two groups (P = 0.171). The 24-h urine Cr level was insignificantly different between the two groups in men (13.5 ± 4.2 mg/kg/day vs. 12.9 ± 4.5 mg/kg/day, P = 0.487) and women (9.8 ± 3.8 mg/kg/day vs. 10.0 ± 2.8 mg/kg/day, P = 0.873). Regarding the association between the difference in the location of urine collection and the complete urine collection, complete urine collections were observed in 45 out of the 131 inpatients (34.4%) and 13 out of the 44 outpatients (29.5%). There was no significant difference between the two groups (34.4% vs. 29.5%, P = 0.585). The mean 24-h urine Na in the inpatient group was lower than that in the outpatient group (87.5 ± 69.5 mmol vs. 162.5 ± 82.4 mmol, P<0.001). The proportion of greater than 78 mmol/day of 24-h urine Na was lower in the inpatient group than that in the outpatient group (48.9% vs. 86.4%, P<0.001) (Table 3).

**Table 3:** 24-h urine test result and complete urine collection between the inpatient and outpatient groups.

	Inpatient	Outpatient	P
	N = 131	N = 44	
24-h urine volume (mL)	1698.3 ± 923.4	1914.3 ± 884.9	0.171
24-h uNa (mmol/d)	87.5 ± 69.5	162.5 ± 82.4	0.000
24-h uCr (mg/kg/day)			
All patients	12.6 ± 4.5	12.3 ± 4.4	0.712
Male	13.5 ± 4.2	12.9 ± 4.5	0.487
Female	9.8 ± 3.8	10.0 ± 2.8	0.873
24-h complete urine collection^†^			
All patients	45/131 (34.4%)	13/44 (29.5%)	0.585
Male	32/102 (31.3%)	9/35 (25.7%)	0.669
Female	13/29 (44.8%)	4/9 (44.4%)	0.984
24-h uNa>78 mmol^†^	64 (48.9%)	38 (86.4%)	0.000
24-h uNa<78 mmol^†^	67 (51.1%)	6 (13.6%)	

Values in table are presented as the mean ± standard deviation. The proportions are presented as percentages for categorical variables.

## Discussion

Considering the assessment of complete urine collection based on 24-h urinary Cr excretion level only in 57 patients (32.5%, 57/175), the objective of this clinical study was not achieved. However, we found that the diagnostic performance of the spot urine Na/K ratio in predicting 24-h urine Na greater than 78 mmol/day in the complete urine collection group was as follows: 90.9% sensitivity and 56.0% specificity, with the spot urine Na/K ratio of 1.0. Moreover, the AUROC curve was 0.874 ± 0.051 (P<0.001). The spot urine Na/K ratio reflects 24-h urine Na, but the AUROC value in this study is lower than those of the previous studies [17–19].

Since 1996, Runyon et al. [20] has reported an abstract on the association between the spot urine Na/K ratio and 24-h urine Na, and several studies reported the diagnostic value of the spot urine Na/K ratio. Three studies published as complete articles showed a small number of cirrhotic patients with ascites (40, 40, and 20 patients) [16, 18, 19]. Considering that previously reported retrospective studies comprised small sample sizes, drawing clear conclusions based on the results of these studies was considered difficult. In a previous study, Runyon et al. measured the excretion of Na and K in spot urine and reported that if the spot urine Na/K ratio was greater than 1, there was a 90%–95% probability that 24-h Na excretion in urine was greater than 78 mmol [20]. Stiehm et al. reported that the spot urine Na/K ratio in cirrhotic patients with ascites had 90% accuracy as compared to the 24-h urine Na excretion [13]. El-Bokl et al. studied 40 liver cirrhotic patients with ascites and reported the diagnostic value of the spot urine Na/K ratio in predicting 24-h urine Na greater than 78 mmol/day. The study showed 93.8% sensitivity and 58.3% specificity with 0.9 AUROC value when the spot urine Na/K ratio was 1 [18]. This value is similar to that of our study. Another prospective study comprising 40 cirrhosis patients showed 95.5% sensitivity, 66.7% specificity, and 0.861 AUROC value when the morning spot urine Na/K ratio was 1 and 95.5% sensitivity, 55.6% specificity, and 0.929 AUROC value when the afternoon spot urine Na/K ratio was 1 [16]. Cholongitas et al., in a study comprising 172 cirrhotic patients, reported 81% sensitivity, 92% specificity, and 0.91 AUROC value when the spot urine Na/K ratio was 0.97. However, this study was published in the form of letters to the editors; hence, the study had some limitations considering that the inclusion and exclusion criteria were not described in detail [17]. Currently, the American Association for the Study of Liver Diseases recommended a cutoff value of 1 for the spot urine Na/K ratio [12]. Moreover, previous studies showed sensitivity of 81%–95.5%, specificity of 58.3%–92%, and AUROC value of 0.861–0.948 when the spot urine Na/K ratio was 1 [16–19]. Spot urine Na/K ratio is being used in actual clinical practice. However, previous studies about urine Na/K ratio have a small number of subjects or have not been well validated. Therefore, in our study, we prospectively studied a large number of participants to validate the spot urine Na/K ratio. Sensitivity, specificity, and AUROC of spot urine Na/K of previous studies’ results were presented in Table 4. Compared with previous studies, our study comprising 57 patients showed similar sensitivity (90.9%) but lower specificity (56.0%) and AUROC value (0.832).

**Table 4:** Sensitivity, specificity, and AUROC of spot urine Na/K ratio in predicting 24-h urine Na excretion of previous studies’ results.

Study	Sample size	Na/K ratio	Sensitivity	Specificity	AUROC
Lee et al.	57	1.0	90.9	56.0	0.832
El-Bokl et al. [18]	40	1.0	93.8	58.3	0.900
Park et al. [16]	40	1.0	95.5	66.7	0.861
Da Silva et al. [19]	20	1.0	88	75	0.948
Cholangitas et al. [17]	172	0.97	81	92	0.910

Abbreviations: AUROC area under receiver operating characteristic curve.

Since the previous studies were based on a small number of patients or were designed retrospectively, our study prospectively studied 175 patients to validate the existing recommendations. However, when urine samples were analyzed, only 24-h urine samples from 57 patients (32.6%) were collected appropriately. The rate of complete urine collection was significantly lower in our study compared to the other studies. Therefore, we analyzed the difference in complete urine collection based on the location of urine collection. The rates of complete urine collection were 34.4% in the inpatient group and 29% in the outpatient group. There was no significant difference between the two groups (34.4% vs. 29%, P = 0.585) (Table 3). Therefore, complete urine collection was not associated with the location of urine collection. Moreover, differences in complete urine collection based on the location of urine collection in men and in women were not observed. However, the outpatient group had higher mean 24-h urine Na level and higher proportion of 24-h urine Na greater than 78 mmol/day than the inpatient group, indicating that low-salt diet was not strictly followed at home. High dietary salt intake evaluation is accurate if the patient’s steady-state dry weight and 24-hour urine Na are evaluated together. Our study’s limitation is that only one 24-hour urine test was performed without assessing dry weight.

In this study, Cr excretion level is used to determine the accuracy of urine collection. A complete urine collection was confirmed based on a 24-h Cr excretion level of 15 mg/kg/day for men and 10 mg/kg/day for women [14, 21]. However, the 24-h urine Cr excretion level was less than the above-established criteria in several patients (66.8%). Thus, we assessed the association between low Cr excretion level and muscle mass loss and sarcopenia in liver cirrhotic patients. Sarcopenia is one of the most common and frequent complications observed in cirrhotic patients [22]. According to a review paper, the prevalence of malnutrition such as sarcopenia in cirrhotic patients is approximately 20% to 60% [23]. Other previous studies show that urine Cr excretion is a useful method to assess the total body muscle mass in the elderly [24]. The measurable parameters of sarcopenia were muscle mass and strength and physical performance. Three imaging techniques were used to measure muscle mass: CT, magnetic resonance imaging, and dual energy X-ray absorptiometry [25]. Because we retrospectively analyzed sarcopenia, we analyzed sarcopenia based on CT scan results within 3 months before and after participating in this study. Another method of diagnosing sarcopenia was by measuring the skeletal muscle index and psoas muscle per height using CT. The PMTH was calculated to determine sarcopenia when PMTH was less than 17.3 mm/m in men and less than 10.4 mm/m in women [15].

In our study, we compared the mean PMTH according to complete urine collection. The mean PMTH was insignificantly different between the two groups in all patients, but the mean PMTH value was significantly higher in men in the complete urine collection group than that in men in the incomplete urine collection group. Furthermore, although there was no statistically significant difference between the two groups, the proportion of sarcopenia in male cirrhotic patients was higher in the incomplete urine collection group than that in male cirrhotic patients in the complete urine collection group. Therefore, further study is required to assess this association using a more accurate method. Additionally, further studies are required to determine whether it is appropriate to use the 24-h urine Cr excretion criteria in cirrhotic patients with sarcopenia.

## Conclusions

In this study, we could not draw a clear conclusion because the expected number of patients with complete urine collection was not achieved. However, the spot urine Na/K ratio was considered beneficial in predicting 24-h urine Na in liver cirrhotic patients. Although our study had lower specificity and AUROC values than those of the previous studies, the sensitivity obtained in this study was similar to those of previous studies, and the spot urine Na/K ratio could be used as an alternative to 24-h urine Na excretion. In this study, the number of patients with complete urine collection assessed by Cr excretion level was low. Therefore, a more convenience method used to assess low-salt diet compliance is required to control ascites in decompensated cirrhotic patients. Moreover, validating the 24-hour complete urine collection criteria for current cirrhotic patients is considered necessary.

## Supporting information

S1 File (XLSX)
